# Thermally Induced Silane Dehydrocoupling: Hydrophobic and Oleophilic Filter Paper Preparation for Water Separation and Removal from Organic Solvents

**DOI:** 10.3390/ma14195775

**Published:** 2021-10-02

**Authors:** Rae Hyung Kang, Dokyoung Kim

**Affiliations:** 1 Department of Biomedical Science, Graduate School, Kyung Hee University, Seoul 02447, Korea; hpohpo2000@hanmail.net; 2 Medical Research Center for Bioreaction to Reactive Oxygen Species and Biomedical Science Institute, School of Medicine, Kyung Hee University, Seoul 02447, Korea; 3 Center for Converging Humanities, Kyung Hee University, Seoul 02447, Korea; 4 Department of Anatomy and Neurobiology, College of Medicine, Kyung Hee University, Seoul 02447, Korea; 5 KHU-KIST Department of Converging Science and Technology, Kyung Hee University, Seoul 02447, Korea

**Keywords:** cellulose paper, octadecylsilane, silane dehydrocoupling, oil-water separation, superhydrophobic

## Abstract

Organic solvents with high purity are essential in various fields such as optical, electronic, pharmaceutical, and chemical areas to prevent low-quality products or undesired side-products. Constructing methods to remove impurities such as water residue in organic solvents has been a significant challenge. Within this article, we report for the first time a new method for the preparation of hydrophobic and oleophilic filter paper (named OCFP), based on thermally induced silane dehydrocoupling between cellulose-based filter paper and octadecylsilane. We comprehensively characterized OCFP using various characterization techniques (FTIR, XPS, XRD, and EDS). OCFP showed super-hydrophobic and oleophilic properties as well as remarkable water separation and removal efficiency (>93%) in various organic solvents with sustained reusability. In addition, the analytical results both before and after filtration of an NMR solvent using OCFP indicated that OCFP has an excellent solvent drying efficiency. This work presents a new strategy for the development of super-hydrophobic cellulose-based filter paper, which has great potential for solvent drying and water separation.

## 1. Introduction

Organic solvents are widely used in various fields, including optical, electronic, pharmaceutical, and chemical industries, and their quality control is directly related to product quality [1,2,3,4,5,6,7,8,9,10,11,12]. Many factors may reduce the purity of organic solvents, and water is considered the most noticeable impurity [13,14,15]. Water residues in organic solvents can lower the quality of the product or generate undesired side-products that could be harmful to organisms [16,17]. For example, tetrahydrofuran (THF) and dimethylsulfoxide (DMSO) have been widely used in chemical reactions within industry and research institutes, but they are miscible and absorb moisture from the air, which induces undesired reactions in products. Therefore, solvent-drying is a key step in solvents for organic-, inorganic-, and nano-based reactions [18,19,20,21].

**Figure 1:**
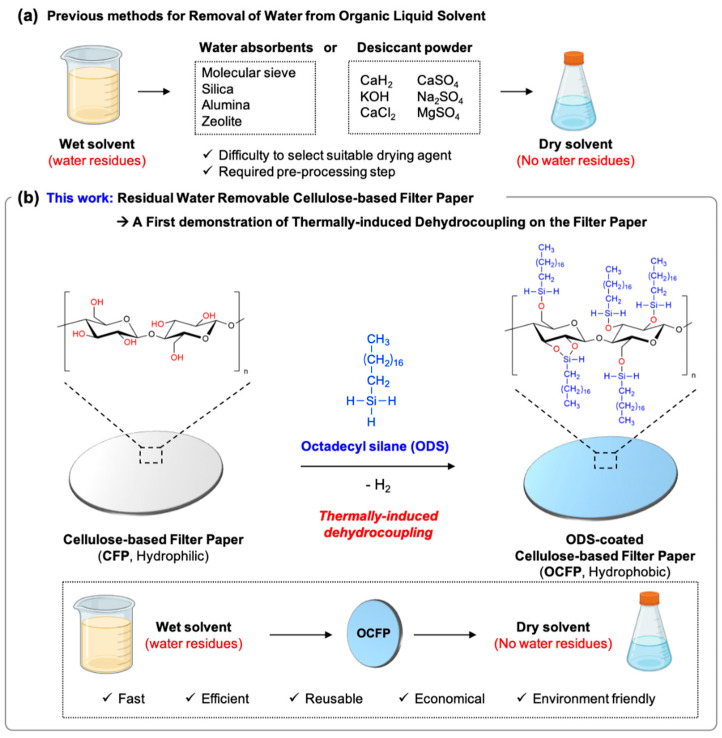
Schematic illustrations of the water removal methods for organic solvents. (**a**) General methods for water removal from organic solvents. (i) Water absorbents such as molecular sieve, silica, alumina, and zeolite. (ii) Desiccant powders such as calcium hydride (CaH_2_), potassium hydroxide (KOH), calcium chloride (CaCl_2_), calcium sulfate (CaSO_4_), sodium sulfate (Na_2_SO_4_), and magnesium sulfate (MgSO_4_). (**b**) Current work: residual water removal cellulose-based filter paper (CFP). The surface of CFP was modified with octadecylsilane (ODS) via a thermally induced dehydrocoupling reaction between the trihydridosilane (Si–H_3_) of ODS and hydrogen oxide (OH) of cellulose-based filter paper (CFP) under mild thermal conditions (80 °C), resulting in obtaining hydrophobic properties.

The conventional method to dry organic solvents is to use drying agents [22,23,24]. Commonly used drying agents are water absorbents such as activated molecular sieve, silica, alumina, zeolite, and desiccant powder including calcium hydride (CaH_2_), calcium sulfate (CaSO_4_), potassium hydroxide (KOH), sodium sulfate (Na_2_SO_4_), calcium chloride (CaCl_2_), and magnesium sulfate (MgSO_4_) (Figure 1a) [24,25]. This method, which uses drying agents, shows a high absorption capacity, is practical, and requires no special apparatus, but it has some limitations: (i) difficulty in selecting suitable drying agents for each solvent, for example, CaCl_2_ is generally not compatible with hydroxy (alcohol, phenol), amino (amine, amide), and carbonyl (acid, ketone, ester) functional groups due to basic impurities such as calcium hydroxide (Ca(OH)_2_) and calcium chloride hydroxide (CaCl(OH)); (ii) non-selective absorption of drying agents: drying agents not only absorb water but also other polar compounds such as alcohol [26]; (iii) need an additional step to remove the drying agents before use.

To make the protocol easier, membrane technology has attracted attention as an efficient technique to separate oil/water mixtures due to its high efficiency and low energy consumption driven by gravitational forces [27,28,29]. Among the potential membrane materials, cellulose has been widely investigated because of its availability, biodegradability, and unique physical, chemical, and mechanical properties [30,31]. In addition to the use of chemically unaltered cellulose chains, the search for new artificial cellulose-based materials prepared by chemical modification of cellulose is an encouraging field of research for altering the physical and chemical properties of native cellulose or preparing functional cellulose-based materials [32,33]. Generally, the modification process to render hydrophobicity to cellulose-based filter paper is categorized into two methods. The first method is surface roughening, which is achieved through etching, sizing of microminerals, and coating with nano- or microparticles [34,35]. However, roughening of the surface, in the first step, requires dip-coating or spray-coating before the etching or particle-coating. This results in complicated chemical composition and post-modifications [36]. The second method is the surface-coating of cellulose with a hydrophobic chemical or polymer [37,38,39,40,41]. The cellulose structure can be modified through several processes, such as spray-coating, dip-coating, polymerization techniques, in situ nanorod or -particle growth, and plasma-etching via chemical vapor deposition. However, they are usually prepared using a multistep process or other special treatments such as electro-spinning and pore generation to endow them with special porous structures and wetting properties. Under these circumstances, there has been a need to develop a new strategy that renders superhydrophobicity to cellulose-based filter paper without complicated post-processes or multiple chemical modification steps, while maintaining its inherent unique physical, chemical, and mechanical properties.

In this study, for the first time, we introduced a simple method for the preparation of hydrophobic and oleophilic filter paper (named OCFP) based on thermally induced silane dehydrocoupling (Figure 1b). OCFP was prepared via a thermally induced silane dehydrocoupling reaction between cellulose-based filter paper (CFP) and octadecylsilane paper under mild reaction conditions without adding any catalyst [42]. We confirmed its high separation efficiency of the residual water from the organic solvents without decomposition of the membrane structure or tensile strength, tensile modulus, or elongation. We fully characterized OCFP by analyzing the chemical composition, surface morphology and porosity, and surface wettability. The oil-water separation efficiency, reusability, and wet solvent drying efficiency of OCFP were also verified with different oil phases and water mixtures using visualized dyes and wet deuterated chloroform (CDCl_3_). This paper reports an adept, effective, and economical hydrophobic modification method for the filter paper, which has the potential for basic research and practical applications throughout various industries.

## 2. Materials and Methods

### 2.1. Materials

Chemical reagents were purchased from Chmlab (Barcelona, Spain), Acros organics (Geel, Belgium), Sigma-Aldrich (St. Louis, MO, USA), Alfa aesar (Haverhill, MA, USA), Merck (Billerica, MA, USA), Samchun pure chemical (Gyeonggi-do, Rep. of Korea), Honeywell (Charlotte, NC, USA), and TCI (Tokyo, Japan). Commercially available reagents and anhydrous solvents were used without further purification. Cellulose filter paper (product No. F1001-070, diameter: 70 mm) was purchased from Chmlab. Thioflavin T (product No. 211760050) was purchased from Acros organics. Oil red O (product No. O0625) was purchased from Sigma-Aldrich. Toluene (anhydrous, 99.8%, product No. 41464) was purchased from Alfa aesar. Dimethyl sulfoxide (product No. 101900) was purchased from Merck. Ethyl alcohol (94.5%, product No. E1095), n-hexane (95.0%, product No. H0114), acetone (99.5%, product No. A0098), and dichloromethane (99.5%, product No. M0822) were purchased from Samchun pure chemical. Chloroform (HPLC grade, product No. AH049) was purchased from Honeywell. Ethylene glycol (product No. E0105) was purchased from TCI.

### 2.2. Instrumentation

Attenuated total reflection Fourier transform infrared (ATR-FTIR) spectroscopy was performed using a Thermo Scientific Nicolet™ iS™ 5 FT-IR spectrometer (64 scans). Scanning electron microscope (SEM) images were obtained and energy dispersive X-ray (EDX) analysis was performed with ultra-high-resolution analytical FE-SEM (SU-70) at the Korea Basic Science Institute (Korea University, Seoul, Korea). For SEM/EDX analysis, samples were prepared by sticking them to conductive tape and coating with a thin layer of Pt for 60 s. The gas adsorption isotherms were measured using an Autosorb-iQ/MP (Quantachrome Inst., Boynton Beach, FL, USA) up to 1 atm of gas pressure. The isotherms were measured at 77 K using highly pure N_2_ (99.999%). Before the nitrogen adsorption experiment, using the Brunnauer-Emmett-Teller (BET) method, the filter papers were degassed under vacuum overnight at 110 °C. Contact angles were measured using a Drop shape analyzer (DSA100, Kruss) and each sample was tested at more than ten different locations. Surface free energy was calculated by using the Owens, Wendt, Rabel, and Kaelble model based on water/glycerol contact angle result [43]. X-ray diffraction (XRD) was analyzed using an X-ray diffractometer (Philips XPERT MPD). X-ray photoelectron spectroscopy (XPS) was analyzed using a high-performance X-ray photoelectron spectrometer (K-Alpha, Thermo Fisher Scientific) at the Korea Basic Science Institute Busan Center (Busan, Korea). Tensile modulus, tensile strength, and elongation at compliance were measured using a universal testing machine (UTM, Testone, Gyeonggi-do, Korea).

### 2.3. Spectroscopic Analysis

UV/vis absorption spectra were recorded using a spectrophotometer (Agilent Technologies Cary 8454, Santa Clara, CA, USA) with a 1 cm standard quartz cell (internal volume of 0.2 mL, Hellma Analytics, Germany). All absorption spectra were obtained at 25 °C.

### 2.4. Preparation of ODS-Coated Cellulose Filter Paper (OCFP)

The cellulose-based filter paper (CFP, 1 piece, diameter: 70 mm) was immersed in a solution of octadecylsilane (ODS, 2 mL, 5.5 mmol) in n-hexane (4 mL) [42]. The reaction was allowed to proceed for 24 h at 80 °C in an 85Φ glass dish while gently shaking it. The ODS-coated cellulose filter paper (OCFP) was then thoroughly washed with n-hexane and dried at 25 °C. The washing step was repeated three times to remove the remaining ODS.

### 2.5. Separation Efficiency of the Oil-Water Mixture Analysis

The oil-water mixtures were prepared by mixing oil (chloroform; CF, dimethyl chloride; DCM, hexane; Hex, toluene; Tol) and deionized water (*v*/*v* = 1:1) using a vortex mixer (600 rpm) for 5 min. The oil red O (0.1 mg/mL) was added to oils, and thioflavin T (0.1 mg/mL) was added to deionized water for UV–Vis absorption spectra analysis of the filtration. The separation experiments were conducted with as-prepared filter paper without any other external forces. A certain volume (5 mL) of the prepared mixtures was poured into the separation equipment right after vortex mixing. The permeation fluxes of the mixtures and the separation efficiency in the filtrate were tested. The filtration velocity was determined using the following Equation (1) [44]. The separation efficiency of the oil-water mixtures was determined using the following Equation (2) [45].

(1) $$\mathrm{Filtration}\;\mathrm{velocity}=\frac{\mathrm{filtrate}\;\mathrm{volume}}{\mathrm{contract}\;\mathrm{area}\times \mathrm{operation}\;\mathrm{time}}$$

(2) $$\mathrm{Separation}\;\mathrm{efficiency}\;(\%)=(1-\frac{\mathrm{ThTfiltrate}}{\mathrm{ThTmixture}})\times 100$$

The concentration of the oil red O and thioflavin T within the filtrate was calculated from the standard absorption curve.

### 2.6. Solvent Drying Efficiency Analysis

^1^H NMR spectra were obtained using an NMR instrument (JEOL JNM 500 MHz). In the NMR spectra, the chemical shifts (δ) are reported in parts per million (ppm) relative to the signal (0.00 ppm), with an internal standard tetramethylsilane (TMS) for the solution in CDCl_3_ (7.26 ppm for 1H). [CDCl_3_]: ^1^H NMR (CDCl_3_, 500 MHz) δ (ppm): 1.59 (s), 7.26 (s); [CDCl_3_ CFP]: ^1^H NMR (CDCl_3_, 500 MHz) δ (ppm): 1.59 (s), 7.26 (s); [CDCl_3_ OCFP]: ^1^H NMR (CDCl_3_, 500 MHz) δ (ppm): 1.59 (s), 7.26 (s).

### 2.7. Statistical Analysis

All data in this manuscript are expressed as the means ± standard deviation. Significance testing was conducted using a two-tailed Student’s *t*-test. Unless otherwise indicated, *p* < 0.05 was considered statistically significant.

## 3. Results and Discussion

### 3.1. Preparation and Characterization of OCFP

**Figure 2:**
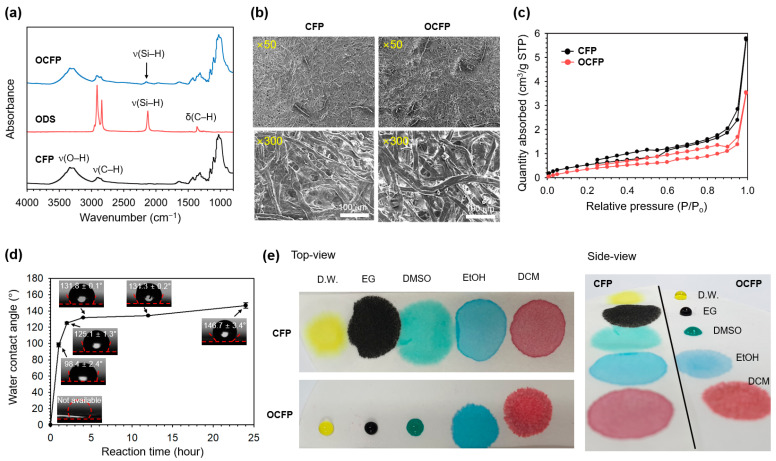
Characterization of ODS-coated cellulose-based filter paper (OCFP). (**a**) Attenuated total reflectance Fourier transform infrared (ATR-FTIR) spectra of the cellulose-based filter paper (CFP), octadecylsilane (ODS), and OCFP. Symbols: ν = stretching and δ = bending. The CFP showed bands associated with the C–OH groups: a broadband for ν(O–H) at 3600‒3000 cm^−1^ and ν(C–H) at 3000–2840 cm^−1^. ODS had two bands at 2100 cm^−1^ associated with ν(Si–H) and at 1380 cm^−1^ derived from the aliphatic chain. (**b**) Scanning electron microscope (SEM) images of the CFP and OCFP. The scale bar is 100 μm. (**c**) Nitrogen adsorption-desorption isotherms for CFP and OCFP. (**d**) Variation in the water contact angle on the surface of OCFP and reaction time. (**e**) Top-view (left) and side-view (right) photographs of the various solvents; deionized water (D.W.), ethylene glycol (EG), dimethyl sulfoxide (DMSO), ethanol (EtOH), and dichloromethane (DCM) on CFP and OCFP.

The octadecylsilane (ODS)-coated cellulose-based filter paper (OCFP) was prepared via a thermally induced silane dehydrocoupling reaction between the hydroxide groups (O–H) of cellulose-based filter paper (CFP) and the trihydridosilane (Si–H_3_) of ODS (Figure 1b). CFP was immersed in a solution of ODS in normal hexane (n-hexane) for 24 h at 80 °C (ODS:n-haxane = 1:2 (*v/v*), optimized ratio). To confirm that CPF and ODS reacted via thermally induced silane dehydrocoupling, we analyzed the surface functional groups of CFP and OCFP (Figure 2a). The attenuated total reflectance Fourier transform infrared (ATR-FTIR) spectrum of CFP displayed two characteristic bands corresponding to ν(O–H) vibration at 3600‒3000 cm^−1^ and ν(C–H) vibration at 3000–2840 cm^−1^, associated with the C–OH groups of the cellulose backbone [46]. ODS showed a distinctive band at 2100 cm^−1^ associated with the ν(Si–H) vibration of trihydridosilane. After the reaction between CFP and ODS, the absorption band at 2100 cm^−1^, corresponding to Si–H groups, confirmed the successful conjugation of ODS with CFP [42]. For calculating the degree of substitution of OCFP, we normalized the intensity of the ν(Si–H) peak and weighed the CFP and OCFP [47]. The weight of CFP (0.346 g) increased to 0.464 g after the reaction. This result indicates that about 0.2 mmol of ODS is modified by 1 mmol of cellulose (degree of substitution: 0.19 ± 0.07). We also investigated the surface functionality of CFP and OCFP using X-ray photoelectron spectroscopy (XPS) (Figure S1 in Supplementary Materials). CFP showed only two photoelectron peaks, C1s at 290.78 eV and O1s at 535.58 eV [48], which indicated that the CFP contained only C and O. In contrast, a new peak at 105.78 eV of Si2p was observed for CFP conjugated with ODS (Figure S1a). The high-resolution XPS spectra of CFP and OCFP were deconvoluted into three regions with C1s (297–278 eV), O1s (524–523 eV), and Si2p (111–92 eV) (Figure S1b–d). Comparing these spectra showed that on the deconvoluted C1s peaks of CPF, there were two peaks at 285.98 and 284.58 eV, and these were assigned to the ether bond (C–O–C) and the carbon–carbon bond (C–C) (Figure S1b,c) [49]. In contrast, a new peak at 102.28 eV appeared, which derived from the silicon bonding in the silicon oxide unit (Si–O) [50], owing to the newly formed silicon monoxide group (Si–O) (Figure S1d). The increased intensity of the C1s signal indicated the successful modification of aliphatic chains (C_18_) of ODS on the surface of the CFP. These results showed the successful reaction of ODS with the hydroxyl groups on the surface of CFP. The XRD results also indicated the successful reaction with the CFP and ODS (Figure S2) [51]. The morphology of CFP and OCFP was investigated by digital photographs and scanning with an electron microscope (SEM) (Figure 2b and Figure S3). In the digital photographs, the shape and color of the CFP and OCFP showed no significant changes. In the magnified surface morphology of SEM, the surface of the CFP did not change after the conjugation step with ODS without pore-clogging or collapsed fiber structures. The composition of the CFP and OCFP was also analyzed by energy-dispersive X-ray spectroscopy (EDX) (Figure S4). After the reaction with ODS, the presence of Si atom was detected, and a slight increase in the atomic C content from 55.27% in the CFP up to 56.10% in the OCFP was observed. This was derived from the attachment of the long alkyl chain of ODS. All these results confirmed the attachment of ODS onto the surface of the CFP through a thermally induced dehydrocoupling silane reaction.

Next, we analyzed the porosity of the CFP and OCFP by nitrogen adsorption-desorption isotherm measurements (Figure 2c and Figure S5). CFP showed a low Barrett-Joyner-Halenda (BJH) surface area (2.097 m^2^/g) containing microsized large pores (Figure 2b, SEM) and nanosized small pores (Figure S5b) with a very low pore volume (0.009 cm^3^/g, Figure S5a). The OCFP showed a slightly decreased surface area and pore volume compared to the CFP, while the pore size increased from 4.887 nm in CFP to 5.626 nm in OCFP. This result indicated that the surface modification of CFP by ODS might reduce the pore size and eventually clog the very small pores (<5 nm). However, OCFP also had porous properties with microsized (Figure 2b) and large nanosized pores, which were mainly distributed between 5 and 30 nm.

The tensile strength of the membrane was measured using a universal test machine (Figure S6, Table S2) for CFP and OCFP. All tests were performed with a specimen size of 30 × 0.16 mm^2^. The results showed that the mechanical strength of the OCFP was similar to that of the CFP, with a very slight decrease in tensile modulus (CFP: 591 MPa, OCFP: 540) and tensile strength (10.78 MPa for OCFP against 11.73 MPa for the CFP) and showed practically the same elongation at compliance (CFP: 3.41%, OCFP: 3.76%). Interestingly, the mechanical properties of the OCFP were maintained after being used once.

### 3.2. Surface Wettability of OCFP

We estimated the water wettability of the OCFP to verify its hydrophobicity (Figure 2d). Before the reaction, the CFP provided no water contact angle value because the water droplet was immediately soaked in CFP. The water contact angle was 91.9° ± 2.4° after 1 h reaction and was dramatically increased to 125.1° ± 1.3° after 2 h of reaction due to the tethering of hydrophobic octadecyl chains. After 24 h of reaction, the water contact angle was further increased to 146.7° ± 3.4° (maximum water contact angle: 150.1°) and remained unchanged. To confirm the accurate hydrophobicity of the OCFP, we measured its surface free energy (Figure S7). The Owens, Wendt, Rabel, and Kaelble model was used for calculation [43]. The surface energy of the OCFP decreased with the reaction time. The surface energy of the OCFP after 1 h reaction was 17.36 ± 8.97 mN/m, and after 24 h of reaction, the surface energy further decreased 1.82 ± 1.26 mN/m. To further investigate the super-hydrophobicity of the OCFP, a various solvent droplet was placed onto the surface of the CFP and OCFP (Figure 2e). While the deionized water (D.W., yellow), ethylene glycol (EG, black), and dimethyl sulfoxide (DMSO, green) droplets were held on the surface of the OCFP and remained nearly spherical with the increase in time, the ethanol (EtOH, blue) and dichloromethane (DCM, red) droplets were absorbed by the OCFP within 1 s. In contrast, the CFP absorbed all solvents. After modification with ODS, the OCFP exhibited super-hydrophobic properties simultaneously, which is highly desirable for water removal from organic solvents.

### 3.3. Separation Efficiency of the Oil-Water Mixtures and Recyclability

**Figure 3:**
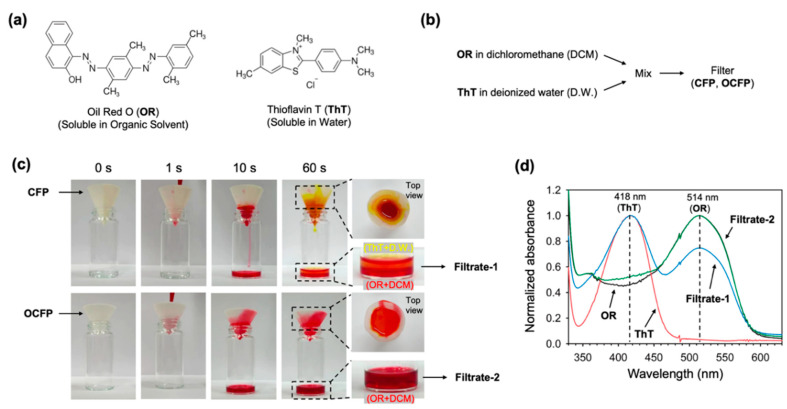
Separation of the oil-water mixture. (**a**) Chemical structures of oil-soluble Oil red O (OR) and water-soluble Thioflavin T (ThT). (**b**) Scheme of the oil-water separation analysis. DCM (with 0.01% Oil red O) and D.W. (with 0.01% Thioflavin T) were mixed using a vortex mixer. (**c**) Separation of the oil-water mixture using CFP and OCFP. (**d**) Normalized UV–Vis spectra of Oil red O (OR, black trace), Thioflavin T (ThT, red trace), filtrate 1 (after filtration of oil-water mixture through CFP, blue trace), and filtrate 2 (after filtration of oil-water mixture through OCFP, green trace).

To evaluate the water separation efficiency of OCFP, the CFP or OCFP was set into a funnel with a circular-cone form to separate organic solvents from a water-containing mixture (Figure 3). The mixture of organic solvent (dichloromethane with 0.1% (*v/v*) oil red O) and water (deionized water with 0.1 (*v/v*) thioflavin T) was poured into the funnel (Figure 3a,b). In the OCFP separation process, dichloromethane permeated rapidly within less than 1 min under the action of gravity by the hydrophobic interaction between the ODS-modified surface and organic solvent, with deionized water (D.W.), and was retained on the funnel due to its superhydrophobic characteristics (Figure 3c, bottom). However, in the CFP separation system, the oil-water mixture permeated through the filter paper without separation. The filtration velocity was evaluated using the mixture [44]. After surface modification, the OCFP filtration velocity was 0.20 mL cm^−^^2^ min^−^^1^, a slight decrease compared to that of the pristine filter paper (0.22 mL cm^−^^2^ min^−^^1^). This result is attributed to the surface modification, which resulted in a small pore size decrease of the filter paper. Next, we investigated the absorption spectra of each filtrate through the CFP and OCFP to measure the remaining ThT or OR in filtrates for the separation efficiency analysis. Thioflavin T (ThT) and oil red O (OR) have characteristic absorption peaks at 418 and 514 nm, respectively (Figure 3d). Filtrate 2, obtained from the DCM and D.W. mixture after filtration through the OCFP, showed only a distinctive absorption peak at 514 nm originating from OR, while filtrate 1, collected from the DCM and D.W. mixture after filtration through CFP, had two characteristic absorption peaks at 418 and 514 nm derived from ThT and OR, respectively. We conducted a separation analysis of the oil (CHCl_3_ and DCM without dye) and water (with yellow dye) mixture to verify the super-hydrophobicity of the OCFP. The result showed that the water did not pass through the OCFP and was on the filter, while oil passed through the OCFP (Figure S8).

**Figure 4:**
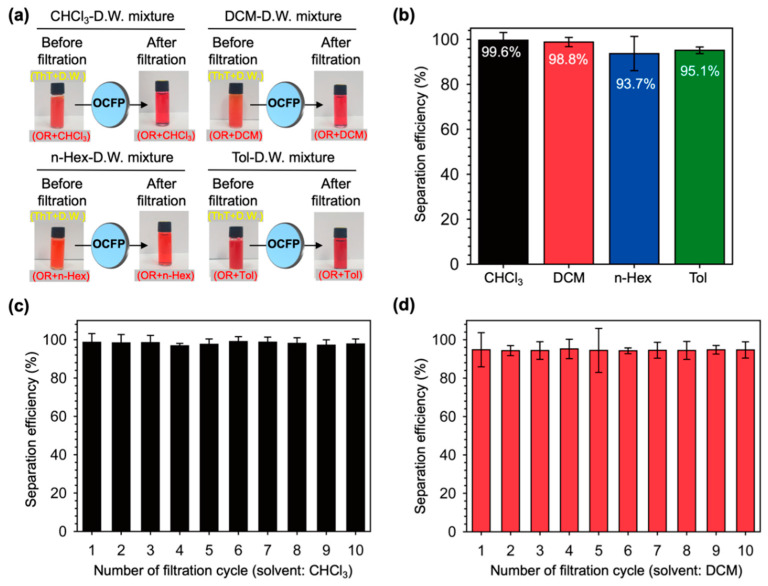
The oil-water separation efficiency of OCFP. (**a**) Representative photographs of wet organic solvents; chloroform (CHCl_3_), dichloromethane (DCM), n-hexane (n-Hex), toluene (Tol) (with 0.01% Oil red O), and D.W. (with 0.01% Thioflavin T) before OCFP filtration (left) and dry organic solvents after OCFP filtration (right). (**b**) The separation efficiency of OCFP for CHCl_3_, DCM, n-Hex, and Tol. Recyclability efficiency of OCFP for (**c**) chloroform (CHCl_3_) and (**d**) dichloromethane (DCM) over 10 cycles.

We further chose three different organic solvents (chloroform, n-hexane, and toluene) to confirm the separation property of OCFP. Before filtration, the oil-water mixture showed orange color because yellow color of ThT and red color of oil red O mixed. After filtration, the filtrates showed the only red color of oil red O and the OCFP consistently exhibited separation efficiency, confirming its excellent organic solvent separation performance (Figure 4a). Th OCFP showed a remarkable separation efficiency higher than 93% for the different organic solvents (chloroform: 99.6%, dichloromethane: 98.8%, hexane: 93.7%, toluene: 95.1%), which indicated that OCFP is effective for removing water from organic solvents (Figure 4a,b).

Next, we checked the stability and reusability of the OCFP. After the first filtration for the mixture separation of the organic solvent (chloroform, dichloromethane) and water, the OCFP was rinsed and dried thoroughly with ethanol and reused again for the same separation. Excellent separation efficiency was maintained even after the OCFP was used for 10 cycles, all higher than 95%, which verified the excellent durability and reutilization of OCFP (Figure 4c,d).

### 3.4. Wet Solvent Drying Efficiency of OCFP

**Figure 5:**
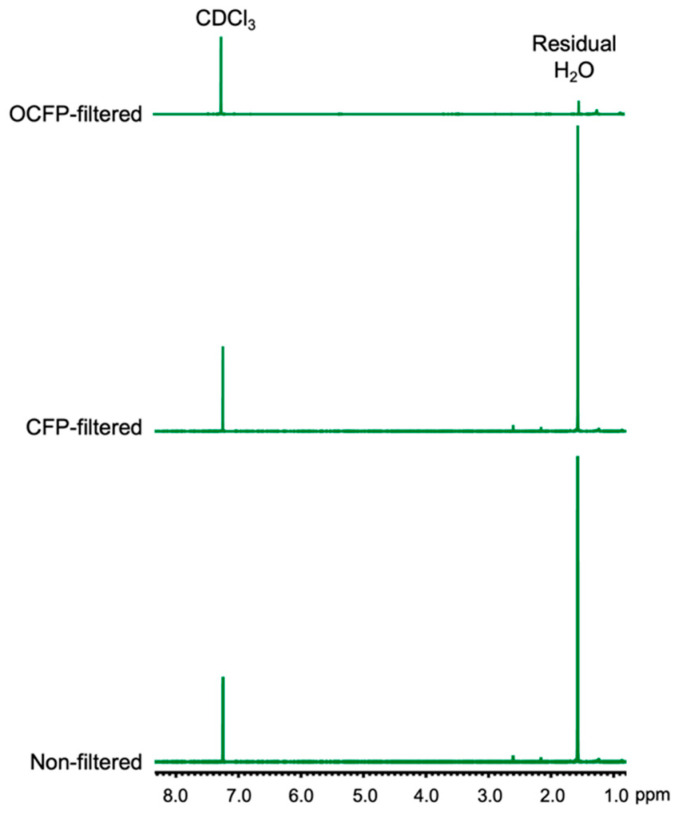
^1^H NMR spectra (500 MHz) of wet deuterated chloroform (CDCl_3_, bottom), CFP-filtered CDCl_3_ (middle), and OCFP-filtered CDCl_3_ (top).

Finally, we examined the wet solvent drying efficiency of the OCFP. We chose deuterated chloroform (CDCl_3_) as a wet solvent and measured the water content in CDCl_3_ using NMR spectroscopy analysis (Figure 5). Commercial CDCl_3_ solvent (non-filtered, stored in a desiccator at 25 °C for 6 months) showed an intensive peak at 1.59 ppm as the H_2_O characteristic peak (Figure 5, bottom). An intensive H_2_O peak was also observed after filtration of CDCl_3_ using CFP (Figure 5, middle), but the OCFP-filtered CDCl_3_ showed negligible intensity, which indicated that OCFP effectively removed the residual water in CDCl_3_ (Figure 5, top).

## 4. Conclusions

In this present work, we introduced a new method for the preparation of cellulose-based hydrophobic and oleophilic filter paper (OCFP) prepared using a thermally induced silane dehydrocoupling reaction. The chemical reaction between CFP and ODS was confirmed using FTIR, XPS, XRD, and EDS analyses. This OCFP showed super-hydrophobicity and oleophilicity with a maximum water contact angle of 150.1° without morphology, porosity, or mechanical strength changes. The water separation and removal efficiency of the OCFP (filtration velocity: 0.20 mL cm^−^^2^ min^−^^1^) were over 93% in various organic solvents and remained unchanged in the 10th recycled use. In addition, the analysis results before and after filtration of NMR solvent using the OCFP, showed that the OCFP exhibited superior solvent drying efficiency. This work presented a new strategy for the development of super-hydrophobic cellulose-based filter paper, which has great potential for solvent drying and water separation. We think it can be applied to various industries that use organic solvents requiring a convenient and efficient drying system.

## Data Availability

The data presented in this study are available in the article or Supplementary Materials.

## Supplementary Materials

The following are available online at https://www.mdpi.com/article/10.3390/ma14195775/s1, Figure S1: X-ray photoelectron spectroscopy (XPS) spectra of CFP and OCFP with the carbon, oxygen, nitrogen, and silicon signal, Figure S2: XRD analysis of CFP and OCFP, Figure S3: Representative photographs of CFP and OCFP, Figure S4: Scanning electron microscopy (SEM) images and elemental (EDX) data of CFP and OCFP, Figure S5: Porous properties of CFP and OCFP, Figure S6: Universal testing machine (UTM) results of CFP, OCFP, and reused OCFP, Figure S7: Variation in surface free energy of OCFP, Figure S8: Separation of the oil-water mixture using OCFP, Table S1: The atomic ratio of C, O, and Si in CFP and OCFP obtained from EDX, Table S2: Mechanical properties of CFP, OCFP, and reused OCFP.

## References

[1] Sugisaki M., Fujiwara M., Kosumi D., Fujii R., Nango M., Cogdell R.J., Hashimoto H. (2010). Comparison of transient grating signals from spheroidene in an organic solvent and in pigment-protein complexes from Rhodobacter sphaeroides 2.4.1. Phys. Rev. B.

[2] Covington A.K., Dickinson T. (1973). Physical Chemistry of Organic Solvent Systems.

[3] Marchetti P., Jimenez Solomon M.F., Szekely G., Livingston A.G. (2014). Molecular separation with organic solvent nanofiltration: A critical review. Chem. Rev..

[4] Peeva L., da Silva Burgal J., Valtcheva I., Livingston A.G. (2014). Continuous purification of active pharmaceutical ingredients using multistage organic solvent nanofiltration membrane cascade. Chem. Eng. Sci..

[5] Buonomenna M., Bae J. (2015). Organic solvent nanofiltration in pharmaceutical industry. Sep. Purif. Rev..

[6] Zuehlsdorff T., Haynes P.D., Hanke F., Payne M., Hine N.D. (2016). Solvent effects on electronic excitations of an organic chromophore. J. Chem. Theory Comput..

[7] Cui Y., Chung T.-S. (2018). Pharmaceutical concentration using organic solvent forward osmosis for solvent recovery. Nat. Commun..

[8] Kim Y., Mittal A., Robichaud D.J., Pilath H.M., Etz B.D., St. John P.C., Johnson D.K., Kim S. (2020). Prediction of Hydroxymethylfurfural Yield in Glucose Conversion through Investigation of Lewis Acid and Organic Solvent Effects. ACS Catal..

[9] Loópez-Salas N., Vicent-Luna J.M., Posada E., Imberti S., Madero-Castro R., Calero S., Ania C.O., Jiménez-Riobóo R., Gutierrez M.C., Ferrer M.L. (2020). Further Extending the Dilution Range of the “Solvent-in-DES” Regime upon the Replacement of Water by an Organic Solvent with Hydrogen Bond Capabilities. ACS Sustain. Chem. Eng..

[10] Salim A.A., Bakhtiar H., Ghoshal S.K., Huyop F. (2020). Customised structural, optical and antibacterial characteristics of cinnamon nanoclusters produced inside organic solvent using 532 nm Q-switched Nd: YAG-pulse laser ablation. Opt. Laser Technol..

[11] Slang S., Palka K., Jancalek J., Kurka M., Vlcek M. (2020). Deposition and characterization of solution processed Se-rich Ge-Se thin films with specular optical quality using multi-component solvent approach. Opt. Mater. Express.

[12] Imura S., Kobayashi T., Tokunaga E. (2021). More Than 50-Fold Enhanced Nonlinear Optical Response of Porphyrin Molecules in Aqueous Solution Induced by Mixing Base and Organic Solvent. Appl. Sci..

[13] Gottlieb H.E., Kotlyar V., Nudelman A. (1997). NMR chemical shifts of common laboratory solvents as trace impurities. J. Org. Chem..

[14] Jacobs P., Dewé W., Flament A., Gibella M., Ceccato A. (2006). A new validation approach applied to the GC determination of impurities in organic solvents. J. Pharm. Biomed. Anal..

[15] Babij N.R., McCusker E.O., Whiteker G.T., Canturk B., Choy N., Creemer L.C., Amicis C.V.D., Hewlett N.M., Johnson P.L., Knobelsdorf J.A. (2016). NMR Chemical Shifts of Trace Impurities: Industrially Preferred Solvents Used in Process and Green Chemistry. Org. Process. Res. Dev..

[16] Iordan H.H., Roger L.D., Grant D.W.A., Inigo G., Stefan M., Tom Z. (2005). Possible Side Reactions Due to Water in Emulsion Polymerization by Late Transition Metal Complexes. 1. Water Complexation and Hydrolysis of the Growing Chain. Inorg. Chem..

[17] National Research Council (US) Safe Drinking Water Committee (1980). Drinking Water and Health.

[18] Kieboom A.P.G., Perrin D.D., Armarego W.L.F. (1988). Purification of Laboratory Chemicals.

[19] Sabane D.G., Mohite M.T., Pratapwar M.N., Patil R.S., Jagtap T.C. (2017). Study of The Effect of Anhydrous Solvent on Methotrexate by uisng UV-Spectrophotometer. Int. J. Chem. Pharm. Anal..

[20] Rubino A., Camellini A., Kriegel I. (2021). Stable solution emission of 2, 3, 5, 6-Tetrafluoro-7, 7, 8, 8-tetracyanoquinodimethane. Opt. Mater..

[21] Honda K., Ono T., Okano K., Miyake R., Dekishima Y., Kawabata H. (2019). Expression of engineered carbonyl reductase from Ogataea minuta in Rhodococcus opacus and its application to whole-cell bioconversion in anhydrous solvents. J. Biosci. Bioeng..

[22] Pahl C., Pasel C., Luckas M., Bathen D. (2011). Adsorptive Water Removal from Organic Solvents in the ppm-Region. Chem. Ing. Tech..

[23] Williams D.B.G., Lawton M. (2010). Drying of Organic Solvents: Quantitative Evaluation of the Efficiency of Several Desiccants. J. Org. Chem..

[24] Burfield D.R., Lee K.-H., Smithers R.H. (1977). Desiccant efficiency in solvent drying. A reappraisal by application of a novel method for solvent water assay. J. Org. Chem..

[25] Furniss B.S., Hannaford A.J., Smith P.W.G., Tatchell A.R. (1989). Vogel’s Textbook of Practical Organic Chemistry.

[26] Burfield D.R., Smithers R.H. (1983). Desiccant efficiency in solvent and reagent drying. 7. Alcohols. J. Org. Chem..

[27] Zularisam A.W., Ismail A.F., Salim R. (2006). Behaviours of natural organic matter in membrane filtration for surface water treatment—A review. Desalination.

[28] Zhu Y., Wang D., Jiang L., Jin J. (2014). Recent progress in developing advanced membranes for emulsified oil/water separation. NPG Asia Mater..

[29] Beshkar F., Salavati-Niasari M., Amiri O. (2020). Superhydrophobic–superoleophilic copper–graphite/styrene–butadiene–styrene based cotton filter for efficient separation of oil derivatives from aqueous mixtures. Cellulose.

[30] Ummartyotin S., Manuspiya H. (2015). A critical review on cellulose: From fundamental to an approach on sensor technology. Renew. Sustain. Energy Rev..

[31] Suhas, Gupta V.K., Carrott P.J.M., Singh R., Chaudhary M., Kushwaha S. (2016). Cellulose: A review as natural, modified and activated carbon adsorbent. Bioresour. Technol..

[32] Jedvert K., Heinze T. (2017). Cellulose modification and shaping—A review. J. Polym. Eng..

[33] Abushammala H., Mao J. (2019). A Review of the Surface Modification of Cellulose and Nanocellulose Using Aliphatic and Aromatic Mono- and Di-Isocyanates. Molecules.

[34] Wang B., Li J., Wang G., Liang W., Zhang Y., Shi L., Guo Z., Liu W. (2013). Methodology for Robust Superhydrophobic Fabrics and Sponges from In Situ Growth of Transition Metal/Metal Oxide Nanocrystals with Thiol Modification and Their Applications in Oil/Water Separation. ACS Appl. Mater. Interfaces.

[35] Nechyporchuk O., Bordes R., Köhnke T. (2017). Wet Spinning of Flame-Retardant Cellulosic Fibers Supported by Interfacial Complexation of Cellulose Nanofibrils with Silica Nanoparticles. ACS Appl. Mater. Interfaces.

[36] Chen Q., de Leon A., Advincula R.C. (2015). Inorganic–Organic Thiol–ene Coated Mesh for Oil/Water Separation. ACS Appl. Mater. Interfaces.

[37] Liu Y., Liu Z., Liu Y., Hu H., Li Y., Yan P., Yu B., Zhou F. (2015). One-Step Modification of Fabrics with Bioinspired Polydopamine@Octadecylamine Nanocapsules for Robust and Healable Self-Cleaning Performance. Small.

[38] Tursi A., De Vietro N., Beneduci A., Milella A., Chidichimo F., Fracassi F., Chidichimo G. (2019). Low pressure plasma functionalized cellulose fiber for the remediation of petroleum hydrocarbons polluted water. J. Hazard. Mater..

[39] Feng X., Shi Y., Liu J., Yang W. (2015). Fabrication of filter paper with tunable wettability and its application in oil-water separation. J. Sol.-Gel Sci. Technol..

[40] Oyola-Reynoso S., Tevis D.I., Chen J., Chang S.B., Çinar S., Bloch J.-F., Thuo M.M. (2016). Recruiting physisorbed water in surface polymerization for bio-inspired materials of tunable hydrophobicity. J. Mater. Chem. A.

[41] Naik V.V., Crobu M., Nenkataraman N.V., Spencer N.D. (2013). Multiple Transmission-Reflection IR Spectroscopy Shows that Surface Hydroxyls Play Only a Minor Role in Alkylsilane Monolayer Formation on Silica. J. Phys. Chem. Lett..

[42] Kim D., Joo J., Pan Y., Boarino A., Jun Y.W., Ahn K.H., Arkles B., Sailor M.J. (2016). Thermally Induced Silane Dehydrocoupling on Silicon Nanostructures. Angew. Chem. Int. Ed..

[43] Owens D., Wendt R. (1969). Estimation of the Surface Free Energy of Polymers. J. Appl. Polym. Sci..

[44] Luo C., Liu Q. (2017). Oxidant-induced high-efficient musselinspired modification on PVDF membrane with superhydrophilicity and underwater superoleophobicity characteristics for oil/water separation. ACS Appl. Mater. Interfaces.

[45] Hong S.K., Bae S., Jeon H., Kim M., Cho S.J., Lim G. (2018). An underwater superoleophobic nanofibrous cellulosic membrane for oil/water separation with high separation flux and high chemical stability. Nanoscale.

[46] Hospodarova V., Singovszka E., Stevulova N. (2018). Characterization of Cellulosic Fibers by FTIR Spectroscopy for Their Further Implementation to Building Materials. Am. J. Analyt. Chem..

[47] Abdul Hadi N., Wiege B., Stabenau S., Marefati A., Rayner M. (2020). Comparison of Three Methods to Determine the Degree of Substitution of Quinoa and Rice Starch Acetates, Propionates, and Butyrates: Direct Stoichiometry, FTIR, and 1H-NMR. Foods.

[48] Belgacem M.N., Czeremuszkin G., Sapieha S., Gandini A. (1995). Surface characterization of cellulose fibres by XPS and inverse gas chromatography. Cellulose.

[49] Tang X., Wang X., Tang C., Ma J., Zhang S., Li Z., Dong F. (2019). PDA-assisted one-pot fabrication of bioinspired filter paper for oil-water separation. Cellulose.

[50] Jensen D.S., Kanyal S.S., Madaan N. (2014). Silicon (100)/SiO_2_ by XPS. Surf. Sci. Spectra.

[51] Marinkovic F.S., Popovic D.M., Jovanovic J.D., Stankovic B.S., Adnadjevic B.K. (2019). Methods for quantitative determination of filler weight fraction and filler dispersion degree in polymer composites: Example of low-density polyethylene and NaA zeolite composite. Appl. Phys. A.

