# Supplementary material for: Thermally Induced Silane Dehydrocoupling: Hydrophobic and Oleophilic Filter Paper Preparation for Water Separation and Removal from Organic Solvents
Source: Materials (Basel). 2021 Oct 2;14(19):5775. doi: 10.3390/ma14195775 (PMC8510372; doi:10.3390/ma14195775)
Supplement: Supplementary file 1 [file materials-14-05775-s001.zip › materials-1366068-supplementary.pdf]

materials

MDPI

Article

Thermally Induced Silane Dehydrocoupling: Hydrophobic and Oleophilic Filter Paper Preparation for Water Separation and Removal from Organic Solvents

Rae Hyung Kang [1,2] and Dokyoung Kim [1,2,3,4,5,*]

[1] Department of Biomedical Science, Graduate School, Kyung Hee University, Seoul 02447, Korea; hpohpo2000@hanmail.net
[2] Medical Research Center for Bioreaction to Reactive Oxygen Species and Biomedical Science Institute, School of Medicine, Kyung Hee University, Seoul 02447, Korea
[3] Center for Converging Humanities, Kyung Hee University, Seoul 02447, Korea
[4] Department of Anatomy and Neurobiology, College of Medicine, Kyung Hee University, Seoul 02447, Korea
[5] KHU-KIST Department of Converging Science and Technology, Kyung Hee University, Seoul 02447, Korea
* Correspondence: dkim@khu.ac.kr

This file includes:

1. Supporting Figures: Figure S1 to S8
2. Supporting Tables: Table S1 to S2

1. Supporting Figures

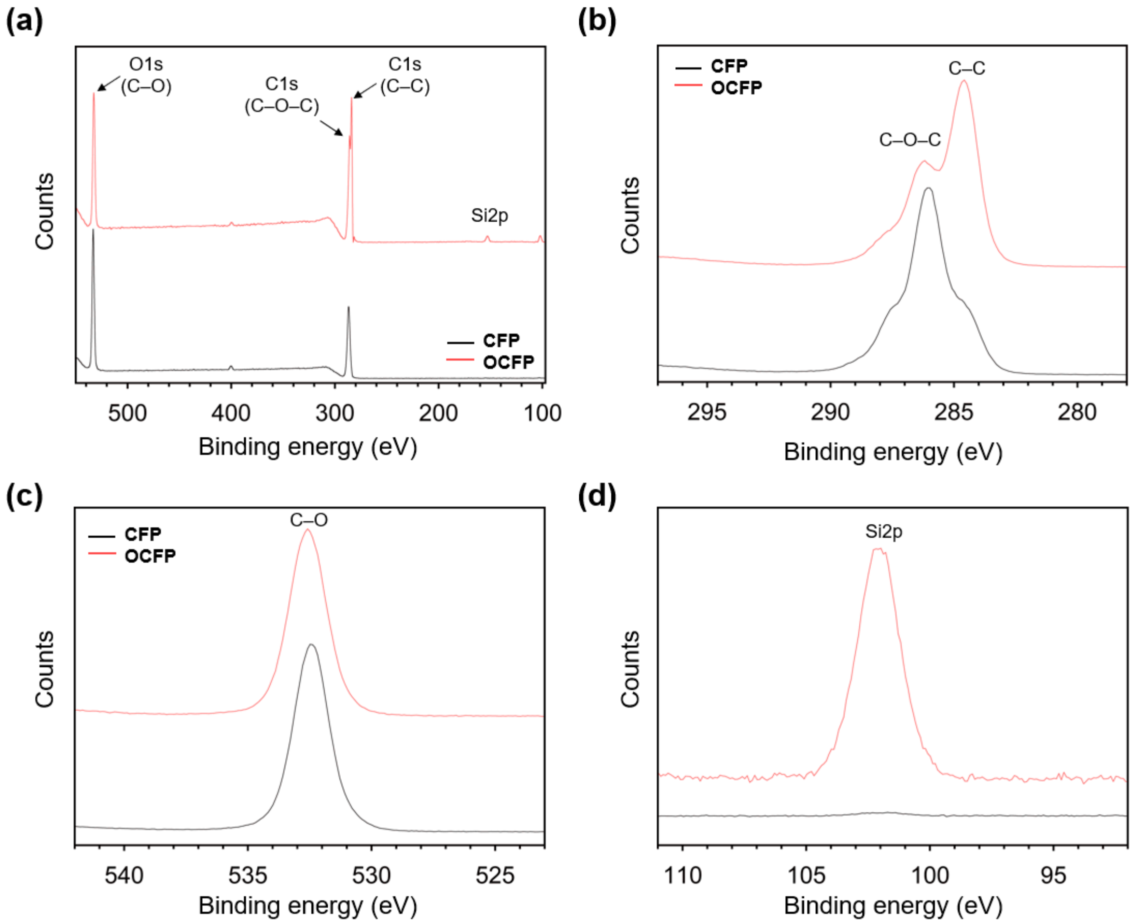

Figure S1. X-ray photoelectron spectroscopy (XPS) spectra of CFP and OCFP with the carbon, oxygen, and silicon signal. (**a**) Survey spectra, (**b**) High-resolution C1s spectra, (**c**) High-resolution O1s spectra, and (**d**) High-resolution Si2p spectra.

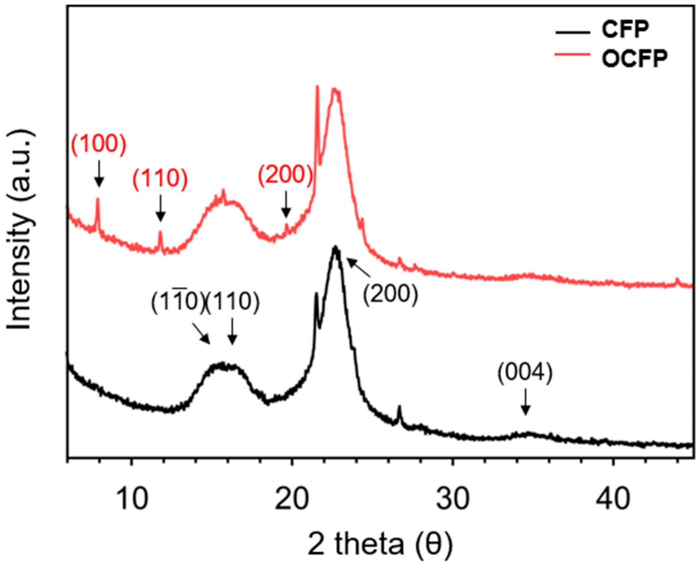

Figure S2. XRD analysis of CFP and OCFP.

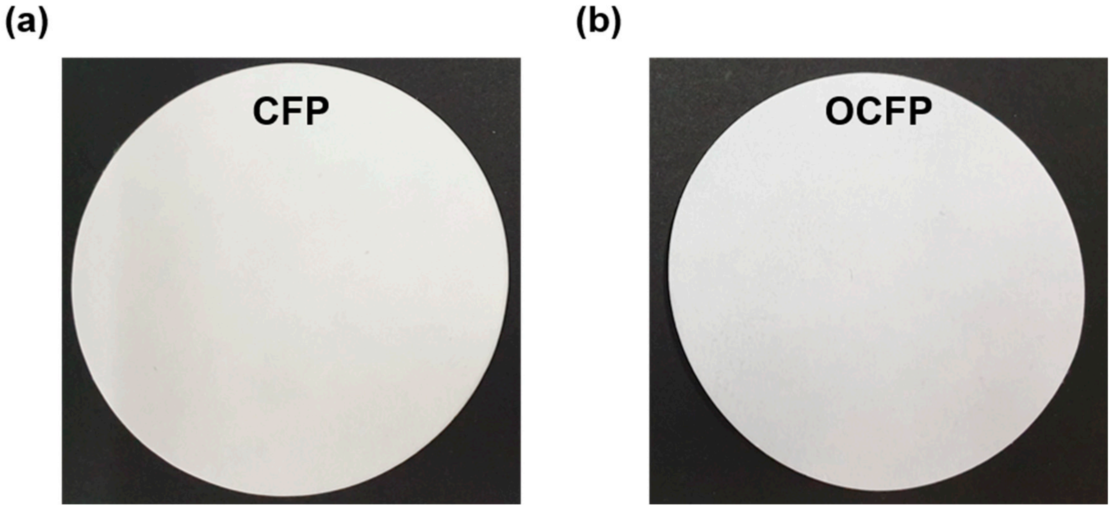

Figure S3. Representative photographs of (**a**) Cellulose-based filter paper (CFP) and (**b**) ODS-coated cellulose-based filter paper (OCFP). The diameter of filter papers is 70 mm.

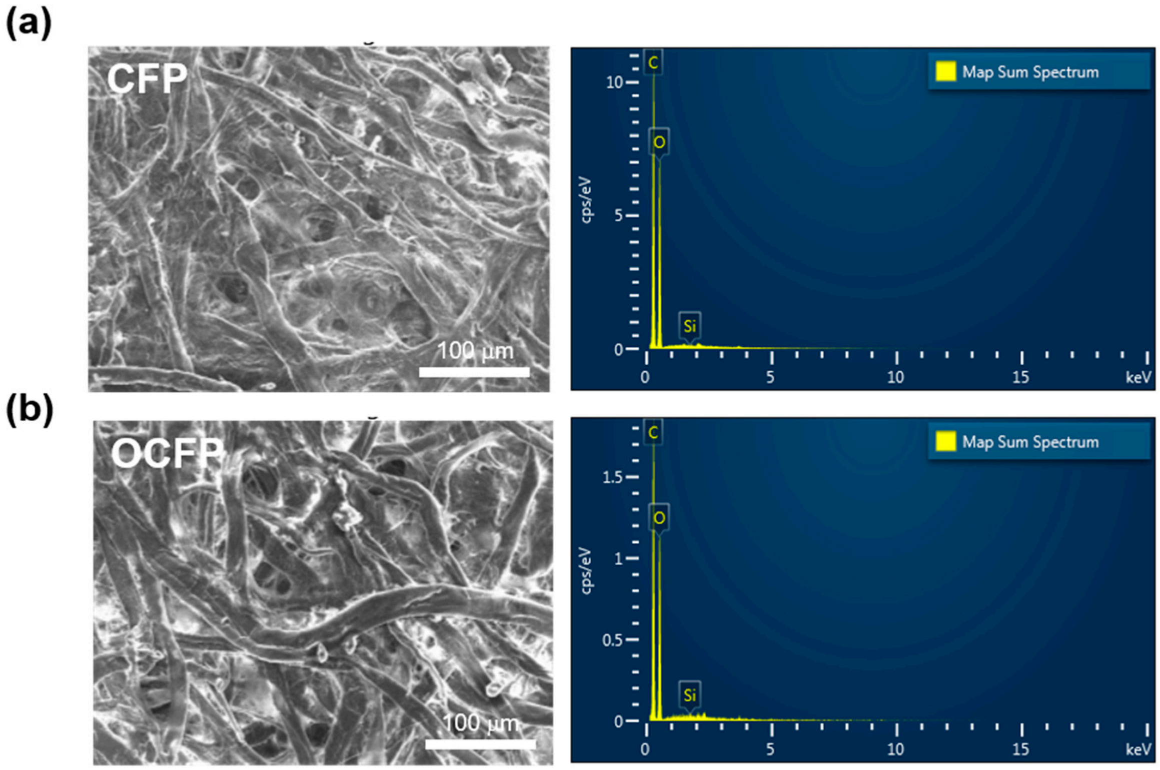

Figure S4. Scanning electron microscope (SEM) images and elemental (EDX) data of (**a**) CFP and (**b**) OCFP. The scale bar is 100 μm.

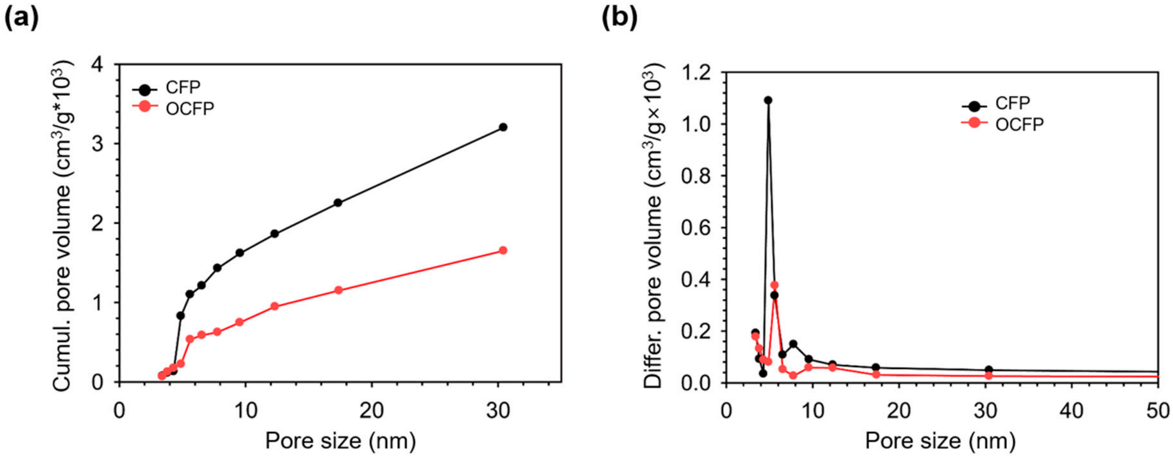

Figure S5. Porous properties of CFP and OCFP. (**a**) Cumulative pore volume and (**b**) Pore size distribution N_2 adsorption rate.

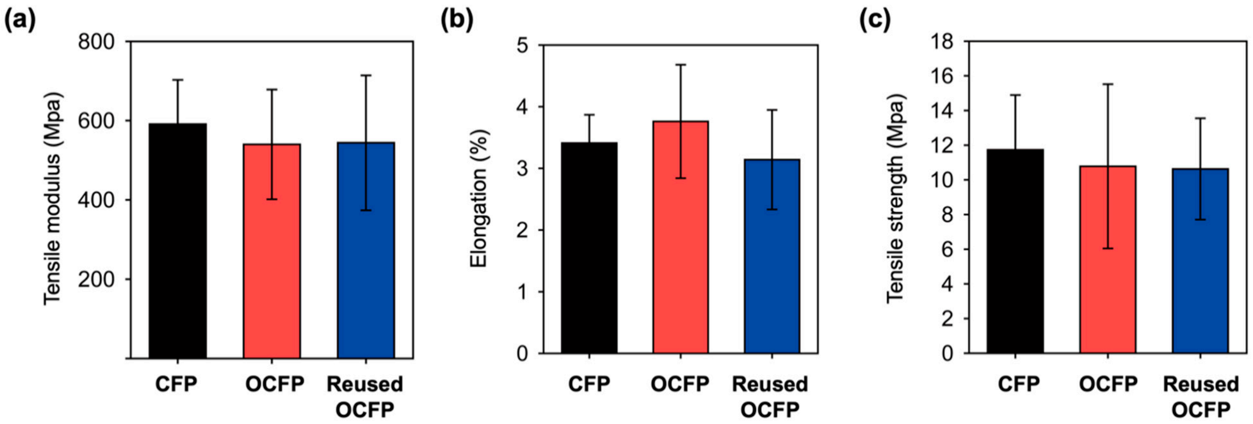

Figure S6. Universal Testing Machine (UTM) results of CFP, OCFP, and reused OCFP. (**a**) Tensile modulus, (**b**) Elongation at compliance, (**c**) Tensile strength.

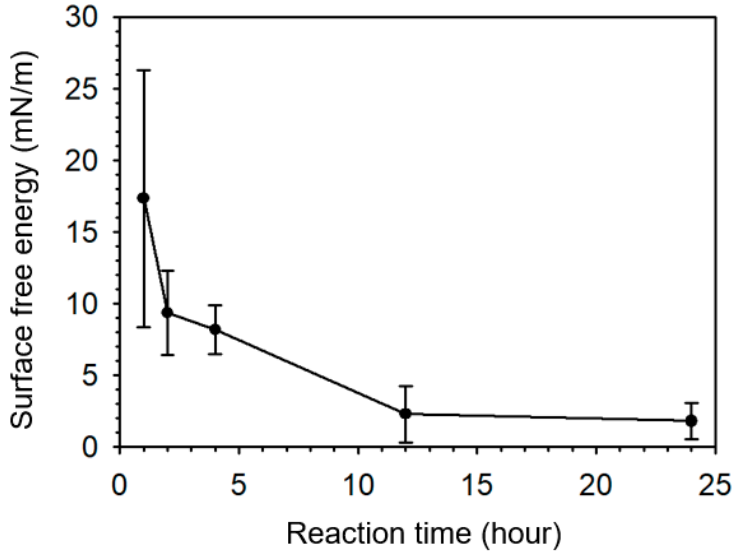

Figure S7. Variation of surface free energy of OCFP as reaction time.

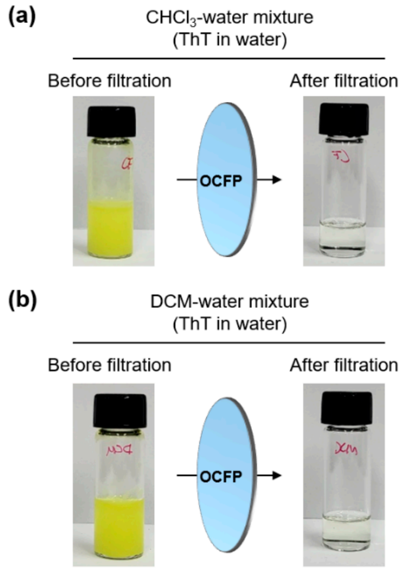

Figure 8. Separation of the oil-water mixture using OCFP. The $CHCl_3$ and DCM mixed with D.W. (with 0.01% ThT).

2. Supporting Tables

Table S1. The atomic ratio of C, O, and Si in CFP and OCFP obtained from EDX.

Sample	C (%)	O (%)	Si (%)	Total (%)
CFP	55.27	44.73	0	100
OCFP	56.10	43.80	0.1	100

Table S2. Mechanical properties of CFP, OCFP, and reused OCFP. All tests were conducted with the specimen size of 30 mm × 0.16 cm (area: 4.80 mm^2).

Sample	Tensile Modulus (MPa)	Elongation at Compliance (%)	Tensile Strength (MPa)
CFP	591	3.41	11.73
OCFP	540	3.76	10.78
Reused OCFP	544	3.14	10.63
